# Long-Term Survival and Risk of Second Malignant Neoplasms Among Childhood Cancer Patients in 16 Provinces of Spain

**DOI:** 10.3390/cancers18121991

**Published:** 2026-06-18

**Authors:** Jaume Galceran, Alberto Ameijide, Noura Jeghalef, Antonia Sánchez, Marcela Guevara, Jàmnica Bigorra, Pilar Gutiérrez, An L. D. Boone, Montserrat Garrido, Xitama Álvarez, Ana Vizcaíno, Isabel Martín, Amaia Onaindia, Silvia Sanclemente, Jan Trallero, María José Sánchez, María-Isabel Palacios, Ramon Clèries, Marià Carulla, on behalf of the Spanish Network of Cancer Registries (REDECAN)

**Affiliations:** 1 Tarragona Cancer Registry, Cancer Epidemiology and Prevention Service, Sant Joan de Reus University Hospital, 43204 Tarragona, Spain; alberto.ameijide@salutsantjoan.cat (A.A.); jamnica.bigorra@salutsantjoan.cat (J.B.); maria.carulla@salutsantjoan.cat (M.C.); 2 Institut de Recerca Biomèdica Catalunya Sud (IRBCatSud), 43204 Reus, Spain; 3 Registry of Childhood and Adolescent Tumours of the Valencian Community, Generalitat Valenciana, 46010 Valencia, Spain; jeghalef_nou@gva.es; 4 Murcia Cancer Registry, Department of Epidemiology, Regional Health Authority, 30008 Murcia, Spain; antonia.sanchez20@carm.es; 5 Instituto Murciano de Investigación Biosanitaria (IMIB)—Arrixaca, 30120 Murcia, Spain; 6 Navarra Cancer Registry, Instituto de Salud Pública y Laboral de Navarra, 31003 Pamplona, Spain; mp.guevara.eslava@navarra.es; 7 Epidemiology and Public Health Area, Navarra Institute for Health Research (IdiSNA), 31008 Pamplona, Spain; 8 CIBER DE Epidemiología y Salud Pública (CIBERESP), 28029 Madrid, Spain; mariajose.sanchez.easp@juntadeandalucia.es; 9 Castilla y León Cancer Registry, Public Health Directorate, Castilla y León Government, 47007 Valladolid, Spain; pilar.gutierrez@jcyl.es; 10 Registro de Tumores de Asturias, Dirección General de Salud Pública y Atención a la Salud Mental, 33005 Oviedo, Spain; anlievedirk.boone@asturias.org; 11 Canary Islands Cancer Registry, Public Health Directorate, Canary Islands Government, 38003 Tenerife, Spain; mcgarmary@gobiernodecanarias.org (M.G.); xalvdia@gobiernodecanarias.org (X.Á.); 12 Castellón Cancer Registry, Directorate General of Public Health, Generalitat Valenciana, 12003 Castellón, Spain; vizcaino_ana@gva.es; 13 Basque Country Cancer Registry, Department of Health, Basque Government, 01010 Vitoria-Gasteiz, Spain; i-martinmunoz@euskadi.eus (I.M.); a-onaindiaagundez@euskadi.eus (A.O.); ms-sanclemente@euskadi.eus (S.S.); 14 Epidemiology Unit and Girona Cancer Registry, Catalan Institute of Oncology, 17190 Girona, Spain; jtrallero@iconcologia.net; 15 Girona Biomedical Research Institute Dr. Josep Trueta (IDIBGI-CERCA), 17190 Girona, Spain; 16 Research Group on Statistics, Econometrics and Health (GRECS), University of Girona, 17004 Girona, Spain; 17 Escuela Andaluza de Salud Pública, 18011 Granada, Spain; 18 Instituto de Investigación Biosanitaria de Granada Ibs.GRANADA, 18012 Granada, Spain; 19 La Rioja Cancer Registry, Servicio de Epidemiología y Prevención Sanitaria, Dirección General de Salud Pública, Consumo y Cuidados, 26071 Logroño, Spain; mipalaciosc@riojasalud.es; 20 Catalan Cancer Plan, Health Department, Catalan Institute of Oncology, 08908 L’Hospitalet de Llobregat, Spain; r.cleries@iconcologia.net; 21 Clinical Sciences Department, Faculty of Medicine, Universitat de Barcelona, 08007 Barcelona, Spain; 22 Bellvitge Biomedical Research Institute (IDIBELL), 08908 L’Hospitalet de Llobregat, Spain

**Keywords:** childhood cancer, incidence, survival, risk, second neoplasms, cancer registry

## Abstract

Background: Childhood cancer survivors have a higher risk of developing malignancies later in life compared with individuals of similar age in the general population. In Spain, population-based evidence on second malignant neoplasms (SMNs) is limited. This study aimed to estimate survival following childhood cancer diagnosis and to assess the risk of SMNs in Spain. Methods: This population-based registry study used data from 12 cancer registries within the Spanish Network of Cancer Registries (REDECAN). All malignancies of sites and non-malignant central nervous system (CNS) tumors diagnosed before age 15 were included. Age-standardized incidence rates (ASIRw) for 2015–2019 were calculated. Five- and 10-year age-adjusted observed survival rates for 1990–1999 and 2000–2009 were estimated. SMN risk was assessed using standardized incidence ratios (SIRs) and excess absolute risks (EARs). Results: The ASIRw for 2015–2019 was 181.3 per million child-years. Ten-year survival increased from 71.3% in 1990–1999 to 75.5% in 2000–2009. The overall risk of developing an SMN was significantly elevated (SIR = 5.67) at 20 years. Conclusions: Although survival for childhood cancer in Spain was around 75% in 2000–2009, childhood cancer survivors remain at substantially increased risk of SMNs for at least two decades. Efforts to reduce treatment-related toxicity while maintaining survival gains are essential.

## Simple Summary

Childhood cancer survival has improved substantially in recent decades; however, survivors remain at increased risk of developing cancer later in life, largely due to treatments received during childhood. This study provides the first population-based analysis in Spain, to (1) evaluate recent trends in childhood cancer survival by tumor type and (2) quantify the short- and medium-term risk of developing second malignant neoplasms (SMNs) according to time since diagnosis and tumor type. Our findings provide valuable evidence to inform strategies aimed at improving survival, reducing long-term treatment-related toxicity, and optimizing long-term follow-up care for survivors.

## 1. Introduction

Childhood cancer survival rates have increased substantially in recent decades largely due to improvements in treatment [1,2]. Currently, five-year survival rates exceed 80% in most European countries, as well as in the United States, Canada, Australia, New Zealand, and several Asian countries [2]. However, childhood cancer survivors often experience long-term adverse health effects [1,2,3] and, among them, an increased risk of developing cancer later in life compared with individuals of similar age in the general population, due to both host factors and cancer treatments [4,5,6,7]. Several studies have shown that the risk of developing a second malignant neoplasm (SMN) within 3 or 5 years after diagnosis is between 3 and 15 times higher than in the general population, and that the cumulative risk at 20 years ranges from 2% to 12% [8,9]. However, this risk may vary over time due to changes in therapeutic strategies. Furthermore, although the main risk factor for a second cancer is treatment, the risk may also vary according to sex, age at first diagnosis, and genetic predisposition [8,9,10]. In the survivor population, the incidence of SMNs does not appear to stabilize over time [11,12,13,14,15].

Because most children have had limited exposure to environmental carcinogens and are expected to survive for many years after treatment, they provide a unique opportunity to study the mechanisms by which chemotherapy agents and radiation may induce carcinogenesis [16]. These considerations highlight the importance of ensuring lifelong follow-up of childhood cancer survivors. Most available evidence is derived from studies restricted to SMNs diagnosed at least 5 years after initial diagnosis [17].

To our knowledge, this study provides the first comprehensive population-based assessment of the risk of SMNs among childhood cancer survivors in Spain. In this study, we present the incidence of childhood cancer for the period 2015–2019, five- and ten-year survival for childhood cancer between 1990 and 2019, and the incidence of second cancers among individuals diagnosed with childhood cancer between 1990 and 2009 in Spain. We also report on the most common SMNs occurring up to 20 years after the initial diagnosis.

## 2. Materials and Methods

### 2.1. Study Design and Data Sources

In this retrospective population-based cohort cancer registry study, all Spanish population-based cancer registries belonging to the Spanish Network of Cancer Registries (REDECAN) were invited to participate. Twelve registries participated, covering 14 provinces and two islands. Three independent analyses were performed: one on the incidence of childhood cancer (0 to 14 years) for the period 2015–2019, another on five- and ten-year survival of childhood cancer for the periods 1990–1999 and 2000–2009, and finally, one on the risk of developing SMNs among individuals diagnosed with childhood cancer during the period 1990–2009 and the sub-periods 1990–1999 and 2000–2009. Table A1 (Appendix A) shows the registries included in each analysis, as well as the corresponding periods covered. In 2000, the proportion of the Spanish child population (ages 0–14) covered by these registries was 31.5%, increasing to 32.2% in 2017. The 0–14 age group represented 14.8% of all ages in 2000 and 14.9% in 2017. The study on the risk of SMNs could only include data from general population registries (of all ages) with data from the period 1990–2009, since it analyzed the risk of secondary neoplasms 20 years after the first cancer diagnosis.

All registries used the 3rd edition of the International Classification of Diseases for Oncology [18] for coding tumor site, morphology, behavior, multiple primary tumors, and basis of diagnosis. Tumors were classified centrally according to the 3rd edition of the International Classification of Childhood Cancer (ICCC-3) [19]. This study includes all malignant tumors of any site, as well as intracranial and intraspinal tumors of benign and uncertain behavior diagnosed before the age of 15 in the study populations.

SMNs were defined as neoplasms occurring at a different anatomical site that did not represent direct spread or metastases of the primary neoplasm, or neoplasms on the same site with a different histological type, and they were selected based on the International Rules for Multiple Primary Cancers of 2004 [20]. Cancers diagnosed after the diagnosis of an SMN were not included in the analysis. SMNs diagnosed within two months of the first cancer were not excluded as it was assumed that most childhood cancers detected shortly after a first diagnosis would have been detected later regardless. Cancer data included individual-level records with information on the following variables: sex, age, date of incidence, tumor sequence (i.e., the numerical order of occurrence of the neoplasm), site, morphology, behavior, most valid basis of diagnosis, data of last follow-up, and vital status.

Population estimates as of July 1 of each year during the study period for each province or island were obtained from the National Institute of Statistics (INE) [21].

Table 1 shows the participating registries, as well as the territory (province/s or island/s) covered by each of them, the population covered in 2000 and in 2017, the number of childhood cancers diagnosed by period, the percentage of cases diagnosed only by death certificate (%DCO) and the percentage of cases with morphological verification (%MV) during the period 2015–2019. DCO cases correspond to tumors of groups I and III. Data were obtained and analyzed from de-identified individual records from the Spanish Network of Cancer Registries (REDECAN) joint database.

### 2.2. Procedures

All cases were extracted from the REDECAN joint database, which is systematically evaluated for data quality. All questionable cases and issues raised were referred to the contributing registries with a request for correction or clarification. This iterative process resulted in an improvement in the overall quality of the data included in the analyses. Table 1 shows two quality indicators for each registry for the period 2015–2019: the proportion of morphologically verified cases and the proportion of cases registered solely from the death certificate.

### 2.3. Statistical Analysis

#### 2.3.1. Incidence Analysis

To estimate the recent incidence of childhood cancer in Spain, we calculated the absolute number of incident cases (cases) and the age-standardized incidence rates (ASIRw) using the World Standard Population [22] for children aged 0–14 years (Table A2—Appendix A) by sex, expressed per million child-years, for the period 2015–2019. We calculated the incidence sex ratios by dividing the number of incident cases in boys by the number of incident cases in girls.

#### 2.3.2. Survival Analysis

We calculated 5-year and 10-year age-adjusted observed survival and their confidence intervals (95% CI), which in children corresponds very closely to relative survival since competing risks of death are negligible. Survival for the periods 1990–1999 and 2000–2009 was estimated from all cases diagnosed during these periods, irrespective of the length of follow-up, using the complete survival approach because almost all children had been followed up for at least 10 years by 31 December 2019. Survival estimates were calculated with the Kaplan–Meier method. In the survival analyses, cases registered solely from the death certificate and cases diagnosed incidentally by autopsy were excluded.

To ensure comparability between sexes and time periods, and with European figures in survival analyses, we standardized according to the age distribution of all European children diagnosed with cancer using the same weights as those used by Botta et al. [23] in four age classes (<1 year, 1–4 years, 5–9 years and 10–14 years) (Table A3—Appendix A).

We calculated survival for all tumors as a whole and for 15 specific categories of ICCC-3: acute lymphoid leukemias (ICCC category Ia), acute myeloid leukemias (Ib), Hodgkin lymphoma (IIa), non-Hodgkin lymphoma except Burkitt lymphoma (IIb), Burkitt lymphoma (IIc), CNS and miscellaneous intracranial and intraspinal neoplasms (III), ependymomas and choroid plexus tumor (IIIa), astrocytomas (IIIb), intracranial and intraspinal embryonal tumors (IIIc), neuroblastoma and ganglioneuroblastoma (IVa), retinoblastoma (V), nephroblastoma and other nonepithelial renal tumors (VIa), osteosarcomas (VIIIa), Ewing tumor and related sarcomas of bone (VIIIc), and rhabdomyosarcomas (IXa). Non-melanoma skin cancers were not included because most cancer registries do not register these malignant neoplasms.

#### 2.3.3. Risk Analysis of Developing a Second Cancer

The standardized incidence ratio (SIR) was calculated as the ratio of the observed number of SMNs to the number expected if patients in the cohort had the same cancer rates as the general reference population. The observed number of cases included all SMNs diagnosed in each cohort (by sex and cancer type) during each defined time period. The expected number of cases was calculated by multiplying the cumulative observed person-years by the incidence rates for cancer site, sex, five-year age group, and calendar year in the general population. The rates of the general population were obtained from the population-based cancer registries that participated in the study.

In each patient, person-years at risk were defined as the period between the first childhood cancer diagnosis and the date of the second cancer diagnosis, the date of death, or the date of end of follow-up, whichever occurred first.

The SIR was calculated by sex, by period of the first diagnosis of childhood cancer (1990–1999, 2000–2009 and 1990–2009) and by time between the first and second cancers (<1 year, 1–4 years, 5–9 years and 10–19 years) for all tumors as a whole. The calculation for two different periods (1990–1999 and 2000–2009) was performed to observe possible differences in risk over time.

The SIR was also calculated based on the type of the first tumor (12 groups of the ICCC-3) for the 20-year period between the first and second cancer diagnoses, by sex.

Finally, the SIR was also calculated based on the type of SMN according to 25 specific ICD-10 categories (lip, oral cavity and pharynx (C00-14), esophagus (C15), stomach (C16), colon (C18), rectum (C19–C21), liver (C22), gallbladder and biliary tree (C23–C24), pancreas (C25), larynx (C32), trachea, lung and bronchus (C33–C34), skin melanoma (C43), breast (C50), cervix uteri (C53), corpus uteri (C54), ovary (C56), prostate (C61), testis (C62), kidney (C64), urinary bladder (C67, D09.0, D41.4), brain and central nervous system (C70–C72), thyroid (C73), Hodgkin lymphoma (C81), non-Hodgkin lymphoma (C82–C86,C88), myeloma (C90) and leukemia (C91–C95)), and an “Other” category covering all remaining malignant tumor types, for the 20-year period between the first and second cancers and by sex. Non-melanoma skin cancers were not included because most cancer registries do not register these malignant neoplasms.

We included all secondary cancers, regardless of the time elapsed between the first and second cancers.

The assumption that the observed number of SMNs follows a Poisson distribution was used to calculate 95% confidence intervals (95% CIs). Results are considered statistically significant if the 95% CI does not include 1.

The excess absolute risk (EAR) was calculated by subtracting the expected number of SMNs from the observed number of SMNs and dividing the difference by the person-years at risk, expressed as the number in excess (or deficit) by 10,000 person-years at risk.

Finally, we calculated the observed age-specific incidence rates of SMNs in the childhood cancer cohort and compared them with the age-specific incidence rates of the general population covered by the cancer registries in Spain.

All the analyses were computed using R software (version 4.5) [24].

## 3. Results

### 3.1. Incidence

A total of 31.56% of the childhood Spanish population aged 0–14 years (contributing 10,942,347 person-years) was covered by the registries included in the incidence calculations for the period 2015–2019 (Table 1 and Table A1—Appendix A). During this quinquennium, 1910 childhood tumors were diagnosed in the population covered by this study. The ASIRw of childhood cancer in Spain during 2015–2019 was 181.3 cases per million person-years, with higher rates observed in boys (192.5) than in girls (170.1). The sex ratio was 1.20. Leukemias were the ICCC-3 group with the highest incidence (53.4), followed by CNS tumors (45.2), lymphomas (23.7), and neuroblastoma (13.1). Table 2 shows the incidence of the 12 ICCC-3 groups by sex.

### 3.2. Survival

**Figure 1:**
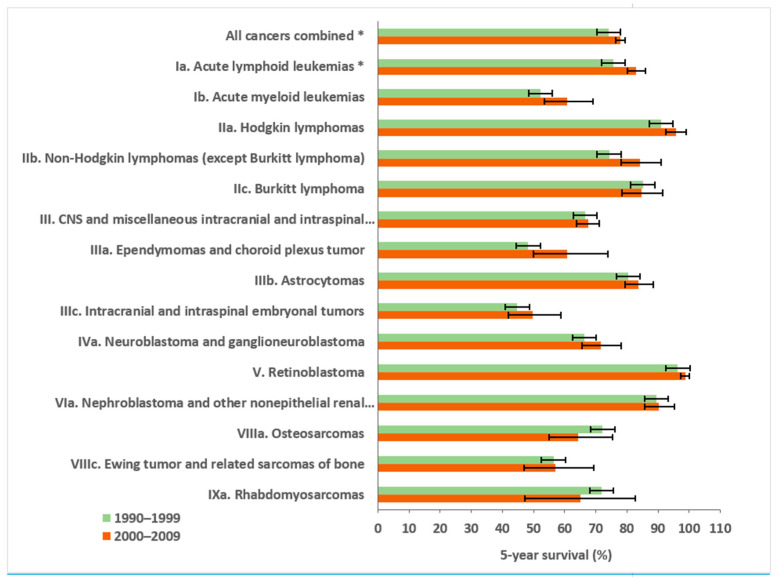
Age-adjusted 5-year observed survival for childhood cancers (0–14 years), all cancers combined and major ICCC-3 groups, for the follow-up periods 1990–1999 and 2000–2009. * statistically significant (*p* < 0.05).

**Figure 2:**
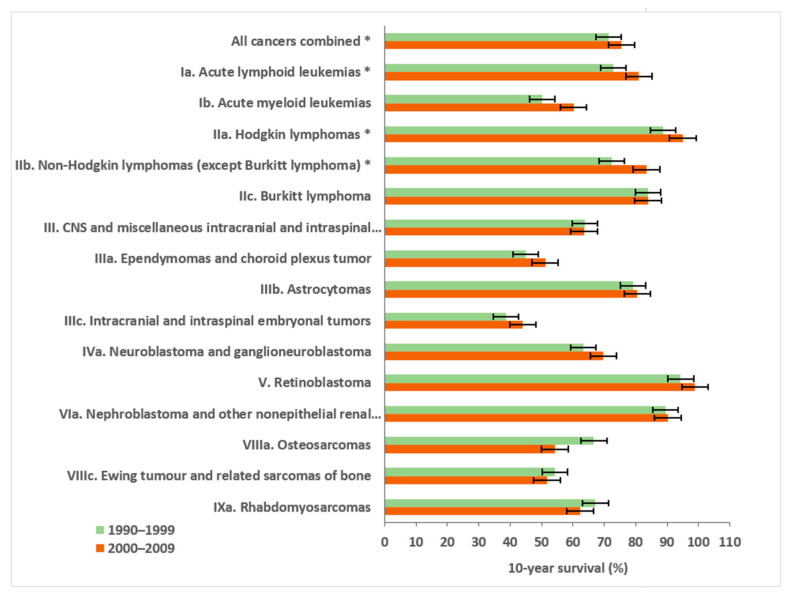
Age-adjusted 10-year observed survival for childhood cancers (0–14 years), all cancers combined and major ICCC-3 groups, for the follow-up periods 1990–1999 and 2000–2009. * statistically significant (*p* < 0.05).

The 5-year age-standardized observed survival rate for all cancers and both sexes combined increased significantly from the period 1990–1999 (74.1; 95% CI: 72.5–75.8) to the period 2000–2009 (77.8; 95% CI: 76.4–79.3). In boys these values were 72.3 and 75.9, and in girls 76.5 and 80.4. The 10-year survival rates also increased between the two periods: 71.3 and 75.5 in both sexes combined, 69.4 and 73.6 in boys, and 73.9 and 78.0 in girls (Table 3 and Figure 1 and Figure 2).

For both sexes combined for the period 2000–2009, the highest 10-year survival rate was observed for retinoblastoma (98.9), followed by Hodgkin lymphomas (95.0), nephroblastoma and other nonepithelial renal tumors (90.3), Burkitt lymphomas (83.9), non-Hodgkin lymphomas (83.5), lymphoid leukemias (81.0) and astrocytomas (80.5). Rates below 80% were observed for neuroblastoma and ganglioneuroblastoma (69.7), CNS and miscellaneous intracranial and intraspinal tumors (63.5), rhabdomyosarcomas (62.3), and acute myeloid leukemias (60.2). The lowest rates corresponded to osteosarcomas (54.3), Ewing tumor and related bone sarcomas (51.7), ependymomas and choroid plexus tumor (51.1), and intracranial and intraspinal embryonal tumors (44.0) (Table 3).

Between 1990–1999 and 2000–2009, 5-year survival rates increased significantly by an average of 3.7 percentage points for all cancers combined. By tumor type, only acute lymphoblastic leukemia showed a significant increase, whereas the remaining cancer types showed non-significant increases (0.6–10.0%), except for three that decreased: Burkitt lymphoma (−0.5%), osteosarcomas (−7.8%), and rhabdomyosarcomas (−6.8%). Between 1990–1999 and 2000–2009, 10-year survival rates increased by an average of 4.2 percentage points for all cancer types combined. Acute lymphoblastic leukemias, Hodgkin lymphomas and non-Hodgkin lymphomas showed significant increases. Across the three most common ICCC-3 groups combined (leukemias, lymphomas, and CNS tumors) survival rates increased by 5.2 percentage points (Table 3).

### 3.3. Risk of Developing a Second Malignant Neoplasm

Table A4 shows the characteristics of childhood cancers included in the second neoplasm risk study, by period. Of the 3834 children under 15 years of age diagnosed with cancer during 1990–2009, 62 (1.6%) developed an SMN during 48,964.01 person-years of follow-up (median follow-up, 15.15 years; interquartile range [IQR], 4.51–20.00 years). The mean time between first and second diagnoses was 12.29 years (IQR, 7.70–18.25 years) for patients diagnosed with a first childhood cancer between 1990 and 1999 and 7.85 years (IQR, 4.11–11.29 years) for patients diagnosed with a first childhood cancer between 2000 and 2009. The mean time between the first and second diagnoses in the second decade was shorter because patients diagnosed in the second decade had a shorter follow-up time (Table A4—Appendix A).

For both sexes combined, the SIR for developing an SMN among individuals with a first childhood cancer diagnosed between 1990 and 2009 was 5.67 at 20 years after first diagnosis. The relative risk was 12.44 during the first year, 5.99 between years 1 and 4, 4.05 between years 5 and 9, and 5.60 between years 10 and 19 after first diagnosis. These values were higher, although not statistically significant, for those diagnosed in 2000–2009 than for those diagnosed in 1990–1999 (Table 4).

Likewise, these risk values were higher, although not statistically significant, in women than in men 20 years after first diagnosis (6.34 versus 5.15), whereas they were lower in women during the first year after first diagnosis (4.51 vs. 17.60). Similar patterns were observed in the ten-year periods of first diagnosis 1990–1999 and 2000–2009 (Table 4).

The EAR 20 years after the first diagnosis of childhood cancer was 10.43 per 10,000 person-years at risk. The excess was higher, although not statistically significant, in women (11.44) than in men (9.60), and in the 2000–2009 period group (12.04) than in the 1990–1999 period group (9.03) (Table 4).

According to age at first cancer diagnosis, all age groups showed significantly elevated SIRs: 6.30 for ages 0–4 years, 5.19 for ages 5–9 years, and 5.47 for ages 10–14 years (Table 5).

The risk of developing an SMN 20 years after the diagnosis of a childhood cancer varied according to the ICCC-3 group of the first childhood cancer (Table 6). Seven ICCC-3 groups showed significantly elevated SIRs: other malignant epithelial neoplasms and malignant melanomas (7.52), CNS tumors (SIR = 7.35), bone tumors (6.87), lymphomas (6.13), soft tissue sarcomas and other extraosseous sarcomas (6.12), neuroblastoma (6.02) and leukemias (4.91). The ICCC-3 groups with the highest EAR per 10,000 person-years were: other malignant epithelial neoplasms and malignant melanomas (18.46), bone tumors (16.63), CNS tumors (14.02), lymphomas (13.56), and soft tissue sarcomas and other extraosseous sarcomas (11.84). By sex, the statistically significant SIRs were higher in women for leukemias (6.28 vs. 3.93), lymphomas (7.87 vs. 5.25), CNS tumors (9.05 vs. 5.74) and other malignant epithelial neoplasms and malignant melanomas, and in men for bone tumors and soft tissue sarcomas and other extraosseous sarcomas (Table 6).

The most common types of SMNs with a statistically significant elevated SIR at 20 years after diagnosis were thyroid carcinomas (n = 12, 19%, SIR = 14.14), leukemias (n = 11, 18%, SIR = 7.07), bone sarcomas (n = 10, 16%, SIR = 16.25), soft tissue sarcomas (n = 6, 10%, SIR = 13.98), brain and central nervous system tumors (n = 5, 8%, SIR = 5.14) and breast carcinomas (n = 3, 5%, SIR = 8.02). By sex, significantly increased SIRs were observed in men for thyroid (33.37), soft tissue (11.73), bone (10.20) and leukemias (8.27), and in women for soft tissue (17.29), breast (8.02), thyroid (7.82) and bone (6.86) (Table 7).

**Figure 3:**
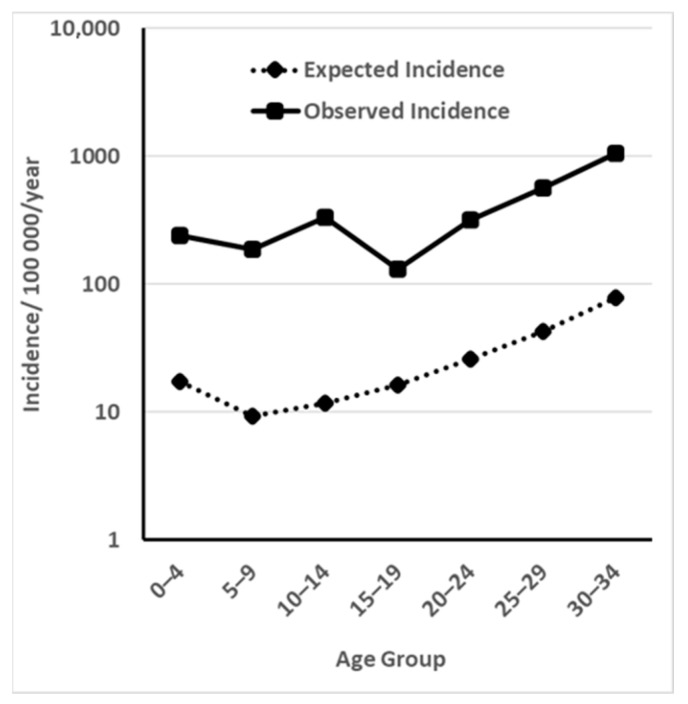
Age-specific observed incidence rates (per 100,000 person-years) of second malignant neoplasms in the study cohort and corresponding incidence rates of the general population covered by cancer registries in Spain.

Finally, Figure 3 shows the age-specific observed incidence rates of SMNs in the study cohort and the corresponding incidence rates in the general population covered by cancer registries in Spain. Across all five-year age groups, the observed rates were clearly higher than expected.

## 4. Discussion

### 4.1. Incidence

Registration of non-malignant CNS tumors may vary across registries, which may lead to a slight underestimation of the incidence of all cancers combined. The ASIRw of childhood cancer for both sexes combined in Spain during the period 2015–2019 was 181.3 per million person-years, a figure slightly higher than that reported by Steliarova-Foucher et al. for Southern Europe during 2001–2010 (170.8) [25]. In our study, ASIRw was higher in boys than in girls, with a sex ratio (SR = 1.13) similar to that reported globally (SR = 1.17) [25]. Sex-specific incidence varied by diagnostic group, with neuroblastomas, germ cell and gonadal tumors, and epithelial tumors being more common in girls than in boys. In Spain, leukemias, followed by CNS tumors, and lymphomas, had the highest ASIRw, consistent with the pattern observed in most regions worldwide, except in Africa, where lymphomas are more common than CNS tumors [25]. The remaining groups followed a similar distribution to that reported for Southern Europe [25] except for bone tumors (group VIII) and other malignant epithelial neoplasms (group XI), which rank seventh and eighth in Southern Europe, compared with eighth and seventh in Spain.

### 4.2. Survival

The five-year survival rate for all childhood cancer combined in Spain during 2000–2009 was 77.8% (95% CI: 76.4–79.3), representing a significant increase of almost four percentage points compared with 1990–1999 (74.1%; 95% CI: 72.5–75.8). Significant progress over time was observed for lymphoid leukemias, the most common childhood cancer. Survival remained stable for the other tumor types, although non-significant increases were observed in most of them, including acute myeloid leukemias, Hodgkin lymphomas, non-Hodgkin lymphomas (except Burkitt lymphomas), CNS tumors, neuroblastomas, retinoblastomas, nephroblastomas, and Ewing sarcomas. Only Burkitt lymphomas, osteosarcomas, and rhabdomyosarcomas showed a decrease in survival rates, although these changes were not statistically significant.

The 5-year survival rate for childhood cancer in Spain during 2000–2009 was 3.5 percentage points lower than that reported for Europe overall and 3.3 percentage points lower than that reported in Spain during 2010–2014 [23], suggesting that survival rates continue to improve. The increase in 5-year survival rates for all childhood cancer combined in Spain between the 1990s and 2000s is similar to that observed in Europe between 2004–2006 and 2010–2014, where rates increased from 78% to 81%. In Europe, significant improvements in survival over time were observed for almost all cancer types, although rates remained stable for Burkitt lymphoma, non-Hodgkin lymphomas, osteosarcomas, Ewing sarcoma, and rhabdomyosarcomas.

Ten-year survival rates also increased significantly between the 1990s and the 2000s. For all tumors combined, the rate rose from 71.3% to 75.5%. The cancer types with the largest increases were lymphoid leukemias (72.8% to 81.0%), Hodgkin lymphomas (88.7% to 95.0%), and non-Hodgkin lymphomas (except Burkitt lymphomas) (72.3% to 83.5%). Although survival for acute myeloid leukemias increased by 10 percentage points (from 50.3% to 60.2%), this change was not statistically significant.

### 4.3. Risk of Developing a Second Malignant Neoplasm

To the best of our knowledge, this is the first study to analyze the risk of developing SMNs after childhood cancer in Spain, using a population-based cohort of 3834 childhood cancer survivors diagnosed between 1990 and 2009, and followed for up to 20 years.

Most studies on secondary neoplasms exclude cancers diagnosed within the first 2 months [15,26], 6 months [27,28], 3 years [29,30,31], or 5 years [16,17,32,33,34,35,36] after the initial diagnosis to minimize selection bias, i.e., the misclassification of progression or recurrence as an SMN. As different studies apply varying exclusion criteria, this decision is inherently subjective. In our study, we included these cancers to estimate the overall risk of developing SMNs, as a substantial proportion of second cancers occur within the first few years after the first diagnosis. Excluding these cases would therefore lead to an underestimation of the absolute risk. Furthermore, the high quality of the registries allowed us to rely on the data regarding SMN. Accordingly, the follow-up period for SMNs began at the date of diagnosis of first malignancy as per Olsen and colleagues in the Nordic countries [7,14]. This methodology provides a more comprehensive estimation of the risk of developing SMNs, regardless of the underlying causes.

The results of this study show an overall SIR of 5.67 for SMNs 19 years after the first cancer diagnosis, with similar values for both sexes. Comparison of SIR values with those reported in other studies is challenging, as they are influenced not only by patient characteristics, environmental factors and treatment of the first cancer, but also by the calendar period of the initial diagnosis, the inclusion criteria for subsequent tumors, the age range at the first diagnosis (0–14 or 0–19 years), the duration of follow-up, the attained age during follow-up, and the study design. These factors partly explain why reported SIRs in childhood cancer survivors vary widely, ranging from 3 to 20 [11,14,17,27,31,32,36,37].

The SIRs for SMNs varied slightly by the calendar period of first childhood cancer diagnosis, with the highest risks observed in children diagnosed during the most recent period (2000–2009) compared with 1990–1999. Although differences in risk were observed across all follow-up intervals (0–, 1–4, 5–9, and 10–19 years), these differences between the two periods were not statistically significant, likely due to the small number of cases. The increase in SIR occurred despite advances in radiotherapy during the 1990s and 2000s; this is consistent with previous findings suggesting a role of chemotherapeutic agents in the etiology of second cancers [14]. Indeed, since the late 1990s, chemotherapy has been intensified, and radiotherapy treatments have been carried out with higher doses in some cancers. In the 1990s, treatments were less intensive, and patients with relapse had poorer survival. Subsequently, relapses began to be actively treated, increasing treatment intensity and, consequently, the risk of SMNs. However, we cannot rule out that part of the observed excess may also reflect improvements in diagnostic and registration procedures (e.g., neuroimaging studies).

Previous studies showed that relative risk of SMNs decreases over time since the first cancer diagnosis, although it remains elevated decades after childhood cancer diagnosis [7,12,15,30,31,32,38]. In our study, the SIR decreased during the first 9 years after the first cancer diagnosis; however, the SIR between 10 and 19 years (5.60) was slightly higher than between 5 and 9 years, although this difference was not statistically significant. This pattern is consistent with the persistence of elevated relative risk over decades, suggesting long-term carcinogenic effects of childhood cancer treatment [14]. Olsen and colleagues also suggested that the observed reduction in relative risk with increasing age may reflect age-related increases in background cancer incidence rather than a true reduction in the carcinogenic effects of childhood cancer treatment.

Conversely, in large studies with long-term follow-up, the EAR attributable to childhood cancer survival status increases over time since the first cancer diagnosis. Therefore, the number of patients with SMNs continues to increase, driven not only by the growing number of long-term survivors, but also by the progressive aging of this population [14]. In our study, although EAR decreased during the first years after childhood cancer, the EAR during the first nine years (7.85; 95% CI: 4.65–12.09) is lower than that observed between 10 and 19 years (14.35; 95% CI: 8.97–21.31).

Young age at first cancer diagnosis has been associated with a higher relative risk of developing SMNs [7,12,15,26,28,31,32,39]. In our study, elevated SIRs were observed across all three age groups. The SIR for children aged 0–4 years (6.30; 95% CI: 3.94–9.55) was only slightly higher than for those diagnosed at ages 5–9 and 10–14 years, although this difference was not statistically significant.

Despite the small number of cases, the SIR for SMNs in our cohort was significantly elevated in seven of the twelve ICCC-3 groups by first cancer type (ranging from 4.91 for leukemias to 7.52 for other malignant epithelial neoplasms and malignant melanomas), and it was greater than 1.0—although not statistically significant—in three additional groups. ICCC-3 Groups I to IV (leukemias, lymphomas, CNS tumors and neuroblastomas) accounted for 69% of the SMNs.

In absolute terms, the most common SMN types according to ICD-10 were thyroid (n = 12), leukemias (n = 11), bone (n = 10), soft tissue (n = 6), central nervous system (n = 5), and breast (n = 3). The highest relative risks were observed for bone sarcomas (SIR = 16.74; 95% CI: 7.74–30.0), thyroid carcinomas (SIR = 14.14; 95% CI: 7.27–24.77), and soft tissue sarcomas (SIR = 13.98; 95% CI: 5.03–30.63). These three tumor types have also been reported to show a high SIR in multiple studies [14]. Significant elevations were also observed for breast cancer, other and unspecified malignancies, leukemias, and CNS and miscellaneous intracranial and intraspinal tumors.

The main strength of this study lies in the use of recent, high-quality population-based data on childhood cancer incidence, survival and the risk of developing SMNs in Spain. The use of high-quality cancer registry data to identify and verify SMNs resulted in a virtually complete and unbiased ascertainment, minimizing potential selection biases due to nonparticipation, loss to follow-up, or differential reporting across treatment centers. In addition, follow-up through data linkage reduced loss to follow-up over time.

One limitation of this study is the relatively small number of cases in some risk categories, which limits a meaningful analysis of certain types of second cancers.

Potentially better ascertainment of patients with SMNs compared with those with a single tumor may have introduced surveillance bias, leading to an overestimation of relative risks. However, screening programs for asymptomatic adult childhood cancer survivors have not been common in Spain; therefore, the impact of this bias is likely to be limited.

Another limitation of the study is that we do not have access to information on SMNs in individuals who were diagnosed with childhood cancer in a region with a population-based registry who subsequently emigrated from that region. This could lead to a slight underestimation of the risk of SMNs.

Finally, for more than 50 years, SMNs in childhood cancer survivors have been recognized as a late sequela of treatment [40], and studies have shown substantial heterogeneity in relative risks according to treatments, with the highest risks observed in patients exposed to both radiotherapy and chemotherapy [31]. A major limitation of this study is the lack of treatment information (e.g., patients who received radiotherapy vs. the ones who did not), which precludes direct assessment of treatment-related risks. Nevertheless, the use of high-quality population-based data provided by cancer registries ensures robust and generalizable estimates. And although we were unable to evaluate them in our study, we believe that radiotherapy (especially for breast and thyroid cancers), some types of chemotherapy (e.g., topoisomerase II inhibitors, alkylating agents) and genetic factors are the main factors in the development of SMNs.

## 5. Conclusions

In the 2000s, the 5-year survival rate for childhood cancer in Spain approached 80%, but survivors remained at high risk of developing second cancers for at least 20 years after the first diagnosis. These findings highlight the need to balance treatment efficacy with long-term safety and to improve survivorship follow-up strategies. Reducing treatment-related toxicity while maintaining survival gains should be a key priority in pediatric oncology.

## Figures and Tables

**Table 1:** Number of childhood cancer cases (0–14 years) and quality indicators by province/island and period.

	Population			Incident Tumors (&)				%DCO	%MV
Registry	Province/Island	2000	2017	1990–1999	2000–2009	2010–2019	2015–2019	2015–2019	2015–2019
Asturias	Asturias	112,979	113,196	182 (&)	172	176 (&)	70 (&)	0.0	95.7
Basque country	Álava/Araba	35,314	48,767	67	77	72	44	0.0	84.1
Basque country	Guipúzcoa/Gipuzkoa	83,378	104,376	158	148	199	103	0.0	96.1
Basque country	Vizcaya/Bizkaia	130,222	153,571	264	236	318	175	1.1	95.4
Canary Islands	Gran Canaria (island)	127,628	114,239	148 (&)	200	203	111	0.0	87.4
Canary Islands	Tenerife (island)	116,242	122,165	125 (&)	172	183	91	0.0	90.1
Castellón	Castellón/Castelló	68,392	87,712	123	107	153	84	1.2	89.3
Castilla y León	Salamanca	43,310	39,091	0	0	72	42	0.0	95.2
Girona	Girona	80,173	123,278	89 (&)	176	211	104	0.0	91.3
Granada	Granada	142,945	141,506	201	218	250	129	0.0	95.3
La Rioja	La Rioja	35,362	46,194	27(&)	59	42	21	0.0	81.0
Murcia	Murcia	204,515	257,748	359	348	468	225	0.0	95.6
Navarra	Navarra	75,198	100,560	131	155	184	90	0.0	94.4
Tarragona	Tarragona	86,242	127,606	156	188	210	103	0.0	86.4
Valencian Community (*)	Alicante/Alacant	222,660	271,751	347	392	421	218	0.5	89.9
Valencian Community (*)	Valencia/València	312,043	383,234	577	610	611	300	0.0	96.0
Total		1,876,604	2,234,994	2831	3258	3773	1910	0.2%	92.8

%DCO: Proportion of cases identified by death certificate only. %MV: Proportion of cases with microscopical verification. (*): Pediatric cancer registry (all other registries are general, covering all ages). (&) Incomplete periods: Asturias: 1991–2017, Canary Islands: 1993–2019, Castellón: 2004–2009, Girona: 1994–2019, La Rioja: 1993–2019, Salamanca: 2011–2019.

**Table 2:** Number of cases and age-standardized incidence rates of childhood tumors (0–14 years), 2015–2019, by sex and main ICCC-3 group.

	Boys		Girls		Total		SR
Tumor Type	n	ASIRw	n	ASIRw	n	ASIRw	
All cancers	1043	192.5	867	170.1	1910	181.3	1.20
I Leukemias, myeloproliferative and myelodysplastic diseases	306	58.5	235	48.3	541	53.4	1.30
II Lymphomas and reticuloendothelial neoplasms	187	31.8	88	15.7	275	23.7	2.13
III CNS and miscellaneous intracranial and intraspinal neoplasms	251	45.4	233	45.0	484	45.2	1.08
IV Neuroblastoma and other peripheral nervous cell tumors	51	11.4	63	14.8	114	13.1	0.81
V Retinoblastoma	21	4.8	23	5.5	44	5.1	0.91
VI Renal tumors	39	8.2	38	8.2	77	8.2	1.03
VII Hepatic tumors	14	2.8	12	2.5	26	2.7	1.17
VIII Malignant bone tumors	45	7.4	42	7.1	87	7.2	1.07
IX Soft tissue and other extraosseous sarcomas	53	9.5	48	8.3	101	8.9	1.10
X Germ cell tumors, trophoblastic tumors and neoplasms of gonads	27	5.0	31	5.7	58	5.4	0.87
XI Other malignant epithelial neoplasms and malignant melanomas	45	7.1	50	8.0	95	7.5	0.90
XII Other and unspecified malignant neoplasms	4	0.7	4	1.0	8	0.8	1.00

Tumors classified according to the International Classification of Childhood Cancer, third edition (ICCC-3). Data are based on the Joint Dataset of the Spanish Network of Cancer Registries (REDECAN). ASIRw: age-standardized incidence rate per million person-years (World Standard Population), ages 0–14 years. SR: sex ratio (boys/girls).

**Table 3:** Five- and 10-year age-adjusted observed survival rates for childhood cancers (0–14 years) by sex and period of diagnosis.

		Boys			Girls		Total		
	n	5-Year (95% CI)	10-Year (95% CI)	n	5-Year (95% CI)	10-Year (95% CI)	n	5-Year (95% CI)	10-Year (95% CI)
1990–1999									
All cancers combined	1576	72.3 (70.1–74.5)	69.4 (67.1–71.7)	1271	76.5 (74.2–78.9)	73.9 (71.5–76.3)	2847	74.1 (72.5–75.8)	71.3 (69.7–73.0)
Ia. Lymphoid leukemias	350	70.6 (65.8–75.8)	67.8 (62.9–73.1)	282	81.9 (77.4–86.7)	79.0 (74.3–84.0)	632	75.7 (72.4–79.3)	72.8 (69.4–76.5)
Ib. Acute myeloid leukemias	63	48.1 (36.3–63.7)	44.3 (33.0–59.5)	67	55.7 (44.6–69.5)	55.7 (44.6–69.5)	130	52.3 (44.0–62.1)	50.3 (42.1–60.1)
IIa. Hodgkin lymphomas	83	91.5 (85.6–97.8)	89.1 (82.7–96.0)	59	86.5 (80.5–93.0)	84.2 (77.1201392.0)	142	91.0 (86.6–95.6)	88.7 (83.7–93.9)
IIb. Non-Hodgkin lymphomas (except Burkitt lymphoma)	75	74.5 (65.3–85.1)	74.5 (65.3–85.1)	34	74.3 (60.9–90.5)	67.0 (52.7–85.3)	109	74.3 (66.6–82.9)	72.3 (64.4–81.1)
IIc. Burkitt lymphoma	93	84.7 (77.5–92.6)	84.7 (77.5–92.6)	22	85.9 (75.0–98.4)	81.3 (68.7–96.2)	115	85.1 (78.6–92.0)	84.0 (77.4–91.2)
III. CNS and miscellaneous intracranial and intraspinal neoplasms	352	63.7 (58.6–69.3)	60.4 (55.2–66.0)	265	70.6 (64.5–77.2)	68.7 (62.6–75.4)	617	66.6 (62.6–70.8)	63.9 (59.9–68.1)
IIIa. Ependymomas and choroid plexus tumor	35	48.6 (32.3–73.1)	43.4 (28.3–66.7)	19	47.8 (29.4–77.8)	47.8 (29.4–77.8)	54	48.3 (35.2–66.4)	44.9 (32.3–62.3)
IIIb. Astrocytomas	153	78.2 (72.0–85.1)	76.9 (70.5–83.9)	124	83.4 (76.5–90.9)	82.5 (75.6–90.1)	277	80.4 (75.6–85.4)	79.2 (74.4–84.4)
IIIc. Intracranial and intraspinal embryonal tumors	75	40.9 (32.1–52.0)	33.6 (25.5–44.3)	43	56.0 (43.4–72.2)	50.9 (38.2–67.9)	118	44.8 (37.6–53.4)	38.7 (31.8–47.2)
IVa. Neuroblastoma and ganglioneuroblastoma	107	62.9 (52.6–75.3)	59.1 (48.7–71.7)	87	70.7 (60.5–82.7)	69.1 (58.9–80.9)	194	66.3 (58.8–74.7)	63.4 (55.7–72.1)
V. Retinoblastoma	33	96.3 (90.9–100.0)	96.3 (90.9–100.0)	32	94.3 (91.1–97.6)	88.8 (80.8–97.5)	65	96.4 (92.9–100.0)	94.4 (90.0–99.0)
VIa. Nephroblastoma and other nonepithelial renal tumors	61	93.3 (88.3–98.7)	93.3 (88.3–98.7)	56	86.2 (78.1–95.1)	86.2 (78.1–95.1)	117	89.5 (84.3–95.0)	89.5 (84.3–95.0)
VIIIa. Osteosarcomas	49	68.2 (56.6–82.3)	61.7 (49.5–76.9)	40	77.3 (65.8–90.9)	72.4 (60.2–87.1)	89	72.2 (63.5–82.2)	66.7 (57.6–77.3)
VIIIc. Ewing tumor and related sarcomas of bone	43	66.3 (53.7–81.9)	64.3 (51.5–80.3)	41	49.4 (37.1–65.7)	47.2 (35.0–63.8)	84	56.4 (46.6–68.2)	54.2 (44.4–66.0)
IXa. Rhabdomyosarcomas	57	74.9 (59.5–90.3)	67.7 (49.5–86.0)	51	69.0 (48.9–89.2)	67.3 (45.1–89.5)	108	71.8 (59.6–84.0)	67.2 (53.3–81.1)
2000–2009									
All cancers combined	1801	75.9 (73.9–77.9)	73.6 (71.6–75.7)	1400	80.4 (78.3–82.5)	78.0 (75.9–80.2)	3201	77.8 (76.4–79.3)	75.5 (74.1–77.1)
Ia. Lymphoid leukemias	404	81.9 (78.0–86.1)	79.5 (75.4–83.8)	302	84.4 (80.3–88.8)	83.3 (79.1–87.8)	706	83.0 (80.1–85.9)	81.0 (78.0–84.0)
Ib. Acute myeloid leukemias	94	58.1 (48.7–69.2)	56.9 (47.6–68.1)	70	65.4 (54.9–78.0)	65.4 (54.9–78.0)	164	60.9 (53.6–69.1)	60.2 (53.0–68.5)
IIa. Hodgkin lymphomas	84	95.5 (91.8–99.4)	95.5 (91.8–99.4)	55	96.9 (93.2–100.0)	95.2 (90.3–100.0)	139	95.8 (92.6–99.0)	95.0 (91.6–98.6)
IIb. Non-Hodgkin lymphomas (except Burkitt lymphoma)	79	84.8 (77.5–92.8)	84.8 (77.5–92.8)	38	85.3 (74.8–97.3)	82.5 (71.0–96.0)	117	84.3 (78.2–90.9)	83.5 (77.2–90.2)
IIc. Burkitt lymphoma	98	84.2 (77.1–91.9)	84.2 (77.1–91.9)	22	89.5 (80.0–100.0)	85.6 (74.2–98.7)	120	84.6 (78.3–91.5)	83.9 (77.4–90.9)
III. CNS and miscellaneous intracranial and intraspinal neoplasms	370	62.8 (57.9–68.1)	58.8 (53.8–64.2)	311	73.2 (68.2–78.6)	69.3 (64.1–74.9)	681	67.5 (63.9–71.2)	63.5 (59.9–67.4)
IIIa. Ependymomas and choroid plexus tumor	53	59.6 (46.2–77.0)	50.5 (37.5–68.1)	27	66.0 (49.4–88.0)	58.7 (41.6–83.0)	80	60.8 (50.0–74.0)	51.1 (40.5–64.6)
IIIb. Astrocytomas	124	80.6 (74.0–87.7)	78.3 (71.4–85.9)	134	86.6 (80.7–92.9)	83.0 (76.7–89.8)	258	83.8 (79.3–88.5)	80.5 (75.7–85.7)
IIIc. Intracranial and intraspinal embryonal tumors	91	42.7 (33.4–54.5)	37.9 (28.9–49.7)	56	60.2 (47.6–76.1)	53.5 (40.7–70.4)	147	49.6 (41.8–58.8)	44.0 (36.4–53.1)
IVa. Neuroblastoma and ganglioneuroblastoma	136	62.1 (53.1–72.5)	60.4 (51.3–71.2)	129	80.2 (72.1–89.2)	78.1 (69.9–87.3)	265	71.6 (65.5–78.2)	69.7 (63.6–76.4)
V. Retinoblastoma	43	100.0 (100.0–100.0)	100.0 (100.0–100.0)	30	97.5 (93.9–100.0)	97.5 (93.9–100.0)	73	98.9 (97.2–100.0)	98.9 (97.2–100.0)
VIa. Nephroblastoma and other nonepithelial renal tumors	69	89.4 (82.6–96.9)	89.4 (82.6–96.9)	76	91.0 (84.9–97.6)	91.0 (84.9–97.6)	145	90.3 (85.6–95.2)	90.3 (85.6–95.2)
VIIIa. Osteosarcomas	47	65.3 (53.4–79.8)	52.9 (40.7–68.9)	38	62.5 (45.6–85.9)	55.5 (39.6–77.7)	85	64.4 (55.0–75.4)	54.3 (44.5–66.3)
VIIIc. Ewing tumor and related sarcomas of bone	49	58.3 (46.0–73.9)	51.8 (39.6–67.9)	28	56.4 (37.4–85.0)	52.6 (34.4–80.5)	77	57.0 (46.9–69.3)	51.7 (41.4–64.5)
IXa. Rhabdomyosarcomas	62	66.5 (46.4–86.6)	63.7 (42.8–84.5)	46	62.2 (42.5–81.8)	59.7 (38.3–81.2)	108	65.0 (47.3–82.7)	62.3 (44.0–80.6)

CI: confidence interval. CNS tumors and all cancers combined: include intracranial and intraspinal tumors of benign and uncertain behavior.

**Table 4:** Risk of developing a second cancer 20 years after childhood cancer diagnosis (1990–2009), by time elapsed since first cancer diagnosis and sex.

	Men			Women			Total		
Time Since First Diagnosis	n	SIR (95% CI)	EAR (95% CI)	n	SIR (95% CI)	EAR (95% CI)	n	SIR (95% CI)	EAR (95% CI)
Period 1990–2009									
0–19 Years	32	5.15 (3.52–7.28 ) *	9.60 (5.83–14.52) *	30	6.34 (4.28–9.06) *	11.44 (7.02–17.27) *	62	5.67 (4.34–7.27) *	10.43 (7.48–14.01) *
<1 year	6	17.60 (6.33–38.55) *	28.32 (9.10–64.09) *	1	4.51 (0.00–25.87)	5.03 (−1.43–35.64)	7	12.44 (4.93–25.78) *	18.16 (6.24–39.33) *
1–4 years	5	4.51 (1.42–10.62) *	5.81 (0.70–15.91) *	6	8.25 (2.97–18.08) *	9.79 (2.66–23.06) *	11	5.99 (2.98–10.76) *	7.59 (3.00–14.83) *
5–9 years	6	4.08 (1.47–8.94) *	5.96 (0.91–15.35) *	4	4.00 (1.04–10.33) *	4.79 (0.06–14.92) *	10	4.05 (1.93–7.47) *	5.43 (1.65–11.53) *
10–19 years	15	4.56 (2.54–7.54) *	11.07 (4.80–20.33) *	19	6.84 (4.11–10.70) *	18.25 (9.72–30.31) *	34	5.60 (3.88–7.84) *	14.35 (8.97–21.31) *
Period 1990–1999									
0–19 Years	13	3.79 (2.01–6.50) *	6.86 (2.48–13.53) *	17	5.95 (3.46–9.55) *	11.49 (5.71–19.84) *	30	4.77 (3.22–6.82) *	9.03 (5.31–13.94) *
<1 year	2	13.01 (1.23–47.83) *	20.08 (0.38–78.34) *	1	8.81 (0.00–50.47)	11.62 (−1.48–73.64)	3	11.22 (2.12–33.22) *	16.24 (1.77–51.20) *
1–4 years	1	2.01 (0.00–11.55)	1.64 (−1.61–17.04)	2	5.42 (0.51–19.93)	6.12 (−0.68–26.20)	3	3.47 (0.65–10.26)	3.72 (−0.52–13.96)
5–9 years	1	1.50 (0.00–8.60)	0.95 (−1.91–14.51)	2	4.03 (0.38–14.82)	4.8 (−0.98–21.88)	3	2.58 (0.49–7.64)	2.77 (−0.90–11.65)
10–19 years	9	4.26 (1.93–8.12) *	10.65 (3.04–23.27) *	12	6.39 (3.29–11.21) *	17.63 (7.48–33.34) *	21	5.26 (3.25–8.06) *	13.93 (7.36–23.07) *
Period 2000–2009									
0–19 Years	19	6.83 (4.11–10.69) *	12.54 (6.68–20.84) *	13	6.95 (3.68–11.91) *	11.38 (5.14–20.89) *	32	6.88 (4.70–9.72) *	12.04 (7.58–17.86) *
<1 year	4	21.36 (5.56–55.24) *	35.34 (7.91–94.14) *	0	0.00 (0.00–36.30)	−1.38 (−1.38–48.67)	4	13.55 (3.52–35.04) *	19.9 (4.00–53.96) *
1–4 years	4	6.54 (1.70–16.92) *	9.35 (1.18–26.86) *	4	11.16 (2.90–28.87) *	13.4 (2.51–36.73) *	8	8.25 (3.52–16.34) *	11.09 (3.86–23.45) *
5–9 years	5	6.23 (1.96–14.65) *	10.2 (1.88–26.62) *	2	3.96 (0.37–14.57)	4.78 (−1.01–21.91)	7	5.35 (2.12–11.09) *	7.86 (2.03–18.22) *
10–19 years	6	5.09 (1.83–11.16) *	11.72 (2.38–29.08) *	7	7.77 (3.08–16.10) *	19.38 (5.95–43.23) *	13	6.25 (3.32–10.72) *	15.04 (6.63–27.84) *

SIR: standardized incidence ratio; EAR: excess absolute risk; 95% CI: 95% confidence interval; * statistically significant (*p* < 0.05).

**Table 5:** Risk of developing a second cancer 20 years after childhood cancer diagnosis (0–14 years, 1990–2009), by age at first cancer diagnosis and sex.

Age	Men			Women			Total		
	n	SIR (95% CI)	EAR (95% CI)	n	SIR (95% CI)	EAR (95% CI)	n	SIR (95% CI)	EAR (95% CI)
0–4	12	5.87 (3.02–10.29) *	8.76 (3.63–16.70) *	10	6.91 (3.29–12.75) *	8.70 (3.37–17.31) *	22	6.30 (3.94–9.55) *	8.73 (4.85–14.09) *
5–9	9	5.89 (2.67–11.23) *	10.58 (3.61–22.14) *	4	4.09 (1.06–10.58) *	5.98 (0.12–18.54) *	13	5.19 (2.75–8.90) *	8.66 (3.62–16.34) *
10–14	11	4.17 (2.07–7.49) *	9.90 (3.34–20.25) *	16	6.95 (3.96–11.31) *	19.01 (9.46–32.94) *	27	5.47 (3.60–7.96) *	14.09 (8.20–21.97) *

SIR: standardized incidence ratio; EAR: excess absolute risk; 95% CI: 95% confidence interval; * statistically significant (*p* < 0.05).

**Table 6:** Risk of developing a second cancer 20 years after childhood cancer diagnosis (0–14 years) by first cancer type and sex.

	Men		Women		Total		
First Cancer	n	SIR (95% CI)	n	SIR (95% CI)	n	SIR (95% CI)	EAR (95% CI)
I-XII. All tumors	32	5.15 (3.52–7.28) *	30	6.35 (4.28–9.07) *	62	5.67 (4.35–7.27) *	10.43 (7.47–14.01) *
I. Leukemias, myeloproliferative and myelodysplastic diseases	7	3.93 (1.56–8.15) *	8	6.28 (2.68–12.43) *	15	4.91 (2.74–8.12) *	8.07 (3.59–14.69) *
II. Lymphomas and reticuloendothelial neoplasms	8	5.25 (2.24–10.40) *	6	7.87 (2.83–17.25) *	14	6.13 (3.34–10.31) *	13.56 (6.18–24.62) *
III. CNS and miscellaneous intracranial and intraspinal neoplasms	4	5.74 (1.49–14.84) *	6	9.05 (3.26–19.84) *	10	7.35 (3.50–13.58) *	14.02 (5.52–27.75) *
IV. Neuroblastoma and other peripheral nervous cell tumors	<3	NC	<3	NC	4	6.02 (1.57–15.57) *	8.39 (0.95–24.35) *
V. Retinoblastoma	<3	NC	0	NC	<3	NC	NC
VI. Renal tumors	<3	NC	0	NC	<3	NC	NC
VII. Hepatic tumors	0	NC	0	NC	0	NC	NC
VIII. Malignant bone tumors	3	7.20 (1.36–21.31) *	<3	NC	5	6.87 (2.17–16.15) *	16.63 (3.31–42.96) *
IX. Soft tissue and other extraosseous sarcomas	3	6.24 (1.18–18.48) *	<3	NC	5	6.12 (1.93–14.41) *	11.84 (2.15–30.97) *
X. Germ cell tumors, trophoblastic tumors and neoplasms of gonads	<3	NC	0	NC	<3	NC	NC
XI. Other malignant epithelial neoplasms and malignant melanomas	<3	NC	4	8.19 (2.13–21.18) *	6	7.52 (2.70–16.47) *	18.46 (4.83–43.82) *
XII. Other and unspecified malignant neoplasms	0	NC	0	NC	0	NC	NC

SIR: standardized incidence ratio; EAR: excess absolute risk; 95% CI: 95% confidence interval; *: statistically significant; NC: not calculated. CNS tumors and all cancers combined: include intracranial and intraspinal tumors of benign and uncertain behavior.

**Table 7:** Risk of developing a second cancer 20 years after childhood cancer diagnosis (0–14 years), by second cancer type and sex.

	Men			Women			Total		
Second Cancer	n	SIR (95% CI)	EAR (95% CI)	n	SIR (95% CI)	EAR (95% CI)	n	SIR (95% CI)	EAR (95% CI)
All. except C44	32	5.15 (3.52–7.28) *	9.59 (5.83–14.51) *	30	6.35 (4.28–9.07) *	11.44 (7.02–17.27) *	62	5.67 (4.35–7.27) *	10.43 (7.47–14.01) *
Lip, oral cavity & pharynx	<3	NC	NC	0	NC	NC	<3	NC	NC
Esophagus	0	NC	NC	0	NC	NC	0	NC	NC
Stomach	<3	NC	NC	0	NC	NC	<3	NC	NC
Colon	0	NC	NC	0	NC	NC	0	NC	NC
Rectum	0	NC	NC	<3	NC	NC	<3	NC	NC
Liver	0	NC	NC	0	NC	NC	0	NC	NC
Gallbladder & biliary tract	0	NC	NC	0	NC	NC	0	NC	NC
Pancreas	0	NC	NC	0	NC	NC	0	NC	NC
Larynx	0	NC	NC	0	NC	NC	0	NC	NC
Lung	0	NC	NC	0	NC	NC	0	NC	NC
Bone	4	10.20 (2.65–26.38) *	1.34 (0.24–3.70) *	6	6.86 (9.67–58.84) *	2.62 (0.88–5.85) *	10	16.25 (7.74–30.00) *	1.92 (0.85–3.64) *
Skin melanoma	0	NC	NC	<3	NC	NC	<3	NC	NC
Soft tissue	3	11.73 (2.21–34.73) *	1.02 (0.12–3.21) *	3	17.29 (3.26–51.19) *	1.28 (0.18–3.94) *	6	13.98 (5.03–30.63) *	1.14 (0.35–2.60) *
Breast				3	8.02 (1.51–23.74) *	1.19 (0.09–3.85) *	3	7.99 (1.51–23.66) *	0.54 (0.04–1.74) *
Cervix uteri				0	NC	NC	0	NC	NC
Corpus uteri				0	NC	NC	0	NC	NC
Ovary				<3	NC	NC	<3	NC	NC
Prostate	0	NC	NC				0	NC	NC
Testis	0	NC	NC				0	NC	NC
Kidney	<3	NC	NC	0	NC	NC	<3	NC	NC
Urinary bladder	0	NC	NC	<3	NC	NC	<3	NC	NC
Brain & CNS	3	5.18 (0.98–15.35)	0.90 (0.00–3.09)	<3	NC	NC	5	5.14 (1.62–12.09) *	0.82 (0.12–2.20) *
Thyroid	7	33.37 (13.23–69.15) *	2.53 (0.95–5.32) *	5	7.82 (2.47–18.41) *	1.97 (0.43–5.04) *	12	14.14 (7.27–24.77) *	2.28 (1.09–4.12) *
Hodgkin lymphoma	0	NC	NC	0	NC	NC	0	NC	NC
Non-Hodgkin lymphoma	<3	NC	NC	<3	NC	NC	<3	NC	NC
Myeloma	0	NC	NC	0	NC	NC	0	NC	NC
Leukemias	8	8.27 (3.53–16.37) *	2.62 (0.91–5.53) *	3	5.11 (0.96–15.12)	1.09 (−0.01–3.76)	11	7.07 (3.51–12.70) *	1.93 (0.80–3.72) *
Others	3	7.31 (1.38–21.65) *	0.96 (0.06–3.15) *	<3	NC	NC	5	7.72 (2.44–18.15) *	0.89 (0.19–2.27) *

SIR: standardized incidence ratio; EAR: excess absolute risk; 95% CI: 95% confidence interval; *: statistically significant (*p* < 0.05); NC: not calculated.

## Data Availability

The data supporting this study have been anonymized. Results are available, always in aggregated form, upon request and formal agreement, provided that there are technical and legal guarantees regarding the protection of personal data and the specific permission of the cancer registries concerned.

## Abbreviations

The following abbreviations are used in this manuscript:

SMN	Second malignant neoplasm
ASIRw	Age-standardized incidence rate
SIR	Standardized incidence ratio
EAR	Excess absolute risk
ICCC-3	International Classification of Childhood Cancer, 3rd edition

## Appendix A

**Table A1:** Participating registries and data periods covered for each of the analyses.

Registry	Incidence 2015–2019	Survival 1990–2009	Second Neoplasms 1990–2009
Asturias	2015–2017	1991–1999; 2000–2009	1991–1999; 2000–2009
Canary Islands	2015–2019	1993–1999; 2000–2009	1993–1999; 2000–2009
Castellón	2015–2019	1990–1999; 2000–2009	2004–2009
Salamanca	2015–2019		
Basque country	2015–2019	1990–1999; 2000–2009	1990–1999; 2000–2009
Girona	2015–2019	1994–1999; 2000–2009	1994–1999; 2000–2009
Granada	2015–2019	1990–1999; 2000–2009	1990–1999; 2000–2009
La Rioja	2015–2019	1993–1999; 2000–2009	1993–1999; 2000–2009
Murcia	2015–2019	1990–1999; 2000–2009	1990–1999; 2000–2009
Navarra	2015–2019	1990–1999; 2000–2009	1990–1999; 2000–2009
Tarragona	2015–2019	1990–1999; 2000–2009	1990–1999; 2000–2009
Valencian Community (*)	2015–2019	1990–1999; 2000–2009	

(*) Monographic childhood cancer registry.

**Table A2:** Weights of the World Standard Population for calculating age-standardized incidence rates (ASIRw).

Age Group (Years)	World Standard Population Weights
0–4	12
5–9	10
10–14	9
Total (0–14)	31

**Table A3:** Weights applied for age standardization.

Cancer	Age Group	Weights	No. of Cases
All cancers combined	<1 year	0.106	7620
	1–4 years	0.358	25,615
	5–9 years	0.251	18,043
	10–14 years	0.285	20,467
I(a) Lymphoid leukemias	<1 year	0.030	579
	1–4 years	0.496	9542
	5–9 years	0.292	5605
	10–14 years	0.182	3495
I(b) Acute myeloid leukemias	<1 year	0.132	470
	1–4 years	0.344	1232
	5–9 years	0.221	788
	10–14 years	0.303	1080
II(a) Hodgkin lymphomas	<1 year	0.001	2
	1–4 years	0.050	185
	5–9 years	0.212	778
	10–14 years	0.737	2710
II(b) Non-Hodgkin lymphomas (except Burkitt lymphoma)	<1 year	0.010	30
	1–4 years	0.174	514
	5–9 years	0.341	1007
	10–14 years	0.475	1401
II(c) Burkitt lymphoma	<1 year	0.000	0.00
	1–4 years	0.232	435
	5–9 years	0.456	856
	10–14 years	0.312	586
III CNS and miscellaneous intracranial and intraspinal neoplasms	<1 year	0.077	876
	1–4 years	0.336	3803
	5–9 years	0.333	3781
	10–14 years	0.254	2883
III(a) Ependymomas	<1 year	0.113	172
	1–4 years	0.475	719
	5–9 years	0.225	341
	10–14 years	0.187	284
III(b) Astrocytomas	<1 year	0.062	200
	1–4 years	0.314	1015
	5–9 years	0.317	1026
	10–14 years	0.307	993
III(c) Intracranial and intraspinal embryonal tumors	0 year	0.091	329
	1–4 years	0.358	1300
	5–9 years	0.361	1317
	10–14 years	0.190	689
IV(a) Neuroblastoma and ganglioneuroblastoma	<1 year	0.388	2120
	1–4 years	0.486	2656
	5–9 years	0.102	559
	10–14 years	0.024	133
V Retinoblastoma	<1 year	0.441	872
	1–4 years	0.525	1036
	5–9 years	0.030	60
	10–14 years	0.004	8
VI(a) Nephroblastoma and other nonepithelial renal tumors	<1 year	0.146	591
	1–4 years	0.628	2552
	5–9 years	0.195	792
	10–14 years	0.031	125
VIII(a) Osteosarcomas	<1 year	0.001	1
	1–4 years	0.019	33
	5–9 years	0.250	433
	10–14 years	0.730	1268
VIII(c) Ewing tumor and related sarcomas of bone	<1 year	0.012	19
	1–4 years	0.090	145
	5–9 years	0.297	477
	10–14 years	0.601	964
IX(a) Rhabdomyosarcomas	<1 year	0.076	190
	1–4 years	0.417	1035
	5–9 years	0.295	733
	10–14 years	0.212	528

Weights are expressed as percentages in the EUROCARE-6 pool based on childhood cancer diagnosed between 2006 and 2013.

**Table A4:** Characteristics of childhood cancers included in the second neoplasm risk study, by period.

	1990–1999		2000–2009	
Characteristic	All (n = 1843)	Cases with SMN (n = 30)	All (n = 1991)	Cases with SMN (n = 32)
Sex				
- Male	1004	13	1160	19
- Female	839	17	831	13
Original ICCC-3 diagnosis				
I—Leukemia	563	7	622	8
II—Lymphoma	293	8	303	6
III—Central nervous system tumors	310	4	296	6
IV—Sympathetic nervous system tumors	128	2	181	2
V—Retinoblastoma	43	1	43	0
VI—Renal tumors	79	1	108	0
VII—Hepatic tumors	23	0	24	0
VIII—Bone tumors	125	3	113	2
IX—Soft tissue tumors	130	3	146	2
X—Germ cell and other gonadal tumors	44	1	60	0
XI—Carcinoma & malignant melanoma	87	2	90	4
XII—Other & unspecified malignant tumors	7	0	5	0
Mean age at original diagnosis (years)	6.65	7.43	6.25	7.59
Mean age at diagnosis of SMN (years)	-	19.73	-	15.44
Mean time from original diagnosis to SMN (years)	-	12.29	-	7.85

SMN: second malignant neoplasm. ICCC-3: International Childhood Cancer Classification, 3rd edition.

## References

[1] Hewitt M., Weiner S.L., Simone J.V. (2003). Childhood Cancer Survivorship: Improving Care and Quality of Life.

[2] Allemani C., Di Carlo V., Ssenyonga N., Baloch F.K., Kuehni C., Girardi F., Goić C., Sophiea M.K., Šekerija M., Espinoza-Vallejos C. (2026). Progress towards the WHO Global Initiative for Childhood Cancer target of 60% 5-year survival for all childhood cancers combined, 1990–2019 (CONCORD-4): A Cancer Survival Index derived for 68 countries by analysis of individual records for 613 021 children from 307 population-based cancer registries. Lancet.

[3] Landier W., Bhatia S. (2008). Cancer survivorship: A pediatric perspective. Oncologist.

[4] Meadows A.T., D’Angio G.J., Evans A.E., Harris C.C., Miller R.W., Mike V. (1975). Oncogenesis and other late effects of cancer treatment in children. Radiology.

[5] Meadows A.T., D’Angio G.J., Miké V., Banfi A., Harris C., Jenkin M.D., Schwartz A. (1977). Patterns of second malignant neoplasms in children. Cancer.

[6] Tucker M.A., Meadows A.T., Boice J.D., Stovall M., Oberlin O., Stone B.J., Birch J., Voûte P.A., Hoover R.N., Fraumeni J.F. (1987). Leukemia after therapy with alkylating agents for childhood cancer. J. Natl. Cancer Inst..

[7] Olsen J.H., Garwicz S., Hertz H., Jonmundson G., Langmark F., Lanning M., Lie S.O., Moe P.J., Møller T., Sankila R. (1993). Second malignant neoplasms after cancer in childhood or adolescence: Nordic Society of Paediatric Haematology and Oncology Association of the Nordic Cancer Registries. BMJ.

[8] Green D.M. (2003). Late effects of treatment for cancer during childhood and adolescence. Curr. Probl. Cancer.

[9] Bhatia S., Sklar C. (2002). Second cancers in survivors of childhood cancer. Nat. Rev. Cancer.

[10] Schwartz C.L. (1999). Long-term survivors of childhood cancer: The late effects of therapy. Oncologist.

[11] Friedman D.L., Whitton J., Leisenring W., Mertens A.C., Hammond S., Stovall M., Donaldson S.S., Meadows A.T., Robison L.L., Neglia J.P. (2010). Subsequent neoplasms in 5-year survivors of childhood cancer: The Childhood Cancer Survivor Study. J. Natl. Cancer Inst..

[12] Neglia J.P., Friedman D.L., Yasui Y., Mertens A.C., Hammond S., Stovall M., Donaldson S.S., Meadows A.T., Robison L.L. (2001). Second malignant neoplasms in five-year survivors of childhood cancer: Childhood Cancer Survivor Study. J. Natl. Cancer Inst..

[13] Hijiya N., Hudson M.M., Lensing S., Zacher M., Onciu M., Behm F.G., Razzouk B.I., Ribeiro R.C., Rubnitz J.E., Sandlund J.T. (2007). Cumulative incidence of secondary neoplasms as a first event after childhood acute lymphoblastic leukemia. JAMA.

[14] Olsen J.H., Möller T., Anderson H., Langmark F., Sankila R., Tryggvadóttír L., Winther J.F., Rechnitzer C., Jonmundsson G., Christensen J. (2009). Lifelong cancer incidence in 47,697 patients treated for childhood cancer in the Nordic countries. J. Natl. Cancer Inst..

[15] Youlden D.R., Baade P.D., Green A.C., Valery P.C., Moore A.S., Aitken J.F. (2020). Second primary cancers in people who had cancer as children: An Australian Childhood Cancer Registry population-based study. Med. J. Aust..

[16] Meadows A.T., Friedman D.L., Neglia J.P., Mertens A.C., Donaldson S.S., Stovall M., Hammond S., Yasui Y., Inskip P.D. (2009). Second Neoplasms in Survivors of Childhood Cancer: Findings from the Childhood Cancer Survivor Study Cohort. J. Clin. Oncol..

[17] Reulen R.C., Frobisher C., Winter D.L., Kelly J., Lancashire E.R., Stiller C.A., Pritchard-Jones K., Jenkinson H.C., Hawkins M.M., British Childhood Cancer Survivor Study Steering Group (2011). Long-term risks of subsequent primary neoplasms among survivors of childhood cancer. JAMA.

[18] World Health Organization (2013). International Classification of Diseases for Oncology.

[19] Steliarova-Foucher E., Stiller C., Lacour B., Kaatsch P. (2005). International Classification of Childhood Cancer, third edition. Cancer.

[20] International Association of Cancer Registries (2004). International Rules for Multiple Primary Cancers (ICD-O Third Edition).

[21] Instituto Nacional de Estadística (INE) Estadística del Padrón Continuo. https://www.ine.es.

[22] Segi M., Fujisaku S., Kurihara M., Narai Y., Sasajima K. (1960). The age-adjusted death rates for malignant neoplasms in some selected sites in 23 countries in 1954–1955 and their geographical correlation. Tohoku J. Exp. Med..

[23] Botta L., Gatta G., Capocaccia R., Stiller C., Cañete A., Dal Maso L., Innos K., Mihor A., Erdmann F., Spix C. (2022). Long-term survival and cure fraction estimates for childhood cancer in Europe (EUROCARE-6): Results from a population-based study. Lancet Oncol..

[24] R Core Team (2024). R: A Language and Environment for Statistical Computing.

[25] Steliarova-Foucher E., Colombet M., Ries L.A.G., Moreno F., Dolya A., Bray F., Hesseling P., Shin H.Y., Stiller C.A., IICC-3 contributors (2017). International incidence of childhood cancer, 2001–2010: A population-based registry study. Lancet Oncol..

[26] Odani S., Nakata K., Inoue M., Kato M., Saito M.K., Morishima T., Hashii Y., Hara J., Kawa K., Miyashiro I. (2023). Incidence of second primary cancers among survivors of childhood cancer: A population-based study, Osaka, Japan, 1975–2015. Cancer Sci..

[27] Ju H.Y., Moon E.-K., Lim J., Park B.K., Shin H.Y., Won Y.-J., Park H.J. (2018). Second malignant neoplasms after childhood cancer: A nationwide population-based study in Korea. PLoS ONE.

[28] Hammal D.M., Bell C.L., Craft A.W., Parker L. (2005). Second primary tumors in children and young adults in the North of England (1968–99). Pediatr. Blood Cancer.

[29] Hawkins M.M., Draper G.J., Kingston J.E. (1987). Incidence of second primary tumors among childhood cancer survivors. Br. J. Cancer.

[30] de Vathaire F., Hawkins M., Campbell S., Oberlin O., Raquin M.A., Schlienger J.Y., Shamsaldin A., Diallo I., Bell J., Grimaud E. (1999). Second malignant neoplasms after a first cancer in childhood: Temporal pattern of risk according to type of treatment. Br. J. Cancer.

[31] Jenkinson H.C., Hawkins M.M., Stiller C.A., Winter D.L., Marsden H.B., Stevens M.C.G. (2004). Long-term population-based risks of second malignant neoplasms after childhood cancer in Britain. Br. J. Cancer.

[32] MacArthur A.C., Spinelli J.J., Rogers P.C., Goddard K.J., Phillips N., McBride M.L. (2007). Risk of a second malignant neoplasm among 5-year survivors of cancer in childhood and adolescence in British Columbia, Canada. Pediatr. Blood Cancer.

[33] Frobisher C., Gurung P.M.S., Leiper A., Reulen R.C., Winter D.L., Taylor A.J., Lancashire E.R., Woodhouse C.R.J., Hawkins M.M. (2010). Risk of bladder tumours after childhood cancer: The British Childhood Cancer Survivor Study. BJU Int..

[34] Armstrong G.T., Liu W., Leisenring W., Yasui Y., Hammond S., Bhatia S., Neglia J.P., Stovall M., Srivastava D., Robison L.L. (2011). Occurrence of multiple subsequent neoplasms in long-term survivors of childhood cancer: A report from the childhood cancer survivor study. J. Clin. Oncol..

[35] Turcotte L.M., Liu Q., Yasui Y., Arnold M.A., Hammond S., Howell R.M., Smith S.A., Weathers R.E., Henderson T.O., Gibson T.M. (2017). Temporal Trends in Treatment and Subsequent Neoplasm Risk Among 5-Year Survivors of Childhood Cancer, 1970–2015. JAMA.

[36] Cardous-Ubbink M.C., Heinen R.C., Bakker P.J.M., van den Berg H., Oldenburger F., Caron H.N., Voûte P.A., van Leeuwen F.E. (2007). Risk of second malignancies in long-term survivors of childhood cancer. Eur. J. Cancer.

[37] Ishida Y., Qiu D., Maeda M., Fujimoto J., Kigasawa H., Kobayashi R., Sato M., Okamura J., Yoshinaga S., Rikiishi T. (2016). Secondary cancers after a childhood cancer diagnosis: A nationwide hospital-based retrospective cohort study in Japan. Int. J. Clin. Oncol..

[38] Jazbec J., Ećimović P., Jereb B. (2004). Second neoplasms after treatment of childhood cancer in Slovenia. Pediatr. Blood Cancer.

[39] Garwicz S., Anderson H., Olsen J.H., Døllner H., Hertz H., Jonmundsson G., Langmark F., Lanning M., Möller T., Sankila R. (2000). Second malignant neoplasms after cancer in childhood and adolescence: A population-based case-control study in the 5 Nordic countries. Int. J. Cancer.

[40] Li F.P., Cassady J.B., Jaffe N. (1975). Risk of second tumors of childhood cancer. Cancer.

